# Autonomic changes in Huntington’s disease correlate with altered central autonomic network connectivity

**DOI:** 10.1093/braincomms/fcac253

**Published:** 2022-10-07

**Authors:** Jordan L Schultz, Amanda E Heinzerling, Alivia N Brinker, Lyndsay A Harshman, Vincent A Magnotta, John A Kamholz, Aaron D Boes, Peg C Nopoulos

**Affiliations:** University of Iowa Carver College of Medicine, Department of Psychiatry, Iowa City, IA, USA; University of Iowa Carver College of Medicine, Department of Neurology, Iowa City, IA, USA; University of Iowa College of Pharmacy, Department of Pharmacy Practice and Sciences, Iowa City, IA, USA; University of Iowa Carver College of Medicine, Department of Psychiatry, Iowa City, IA, USA; University of Iowa Carver College of Medicine, Department of Psychiatry, Iowa City, IA, USA; University of Iowa Carver College of Medicine, Department of Pediatrics, Iowa City, IA, USA; University of Iowa College of Medicine, Department of Radiology, Iowa City, IA, USA; University of Iowa Carver College of Medicine, Department of Psychiatry, Iowa City, IA, USA; University of Iowa Carver College of Medicine, Department of Neurology, Iowa City, IA, USA; University of Iowa Carver College of Medicine, Department of Psychiatry, Iowa City, IA, USA; University of Iowa Carver College of Medicine, Department of Neurology, Iowa City, IA, USA; University of Iowa Carver College of Medicine, Department of Pediatrics, Iowa City, IA, USA; University of Iowa Carver College of Medicine, Department of Psychiatry, Iowa City, IA, USA; University of Iowa Carver College of Medicine, Department of Neurology, Iowa City, IA, USA; University of Iowa Carver College of Medicine, Department of Pediatrics, Iowa City, IA, USA

**Keywords:** Huntington’s disease, autonomic dysfunction, central autonomic network

## Abstract

Autonomic dysfunction has been described in patients with Huntington’s disease, but it is unclear if these changes in autonomic tone are related to the central autonomic network. We performed a pilot study to investigate the relationship between the integrity of the central autonomic network and peripheral manifestiations of autonomic dysfunction in premanifest Huntington’s disease. We recruited male participants with pre-motor-manifest Huntington’s disease and a comparison group consisting of healthy, male participants of approximately the same age. As this was a pilot study, only males were included to reduce confounding. Participants underwent a resting-state functional magnetic resonance imaging study to quantify functional connectivity within the central autonomic network, as well as a resting 3-lead ECG to measure heart rate variability with a particular focus on the parasympathetic time-domain measures of root mean square of successive differences between normal heartbeats. The pre-motor-manifest Huntington’s disease participants had significantly decreased root mean square of successive differences between normal heartbeats values compared with the healthy comparison group. The pre-motor-manifest Huntington’s disease group had significantly lower functional connectivity within the central autonomic network, which was positively correlated with root mean square of successive differences between normal heartbeats. Patients with pre-motor-manifest Huntington’s disease have reduced functional connectivity within the central autonomic network, which is significantly associated with observed changes in autonomic function.

## Teaser

Schultz *et al*. report that autonomic dysfunction in patients with presymptomatic Huntington’s disease was associated with decreased functional connectivity within the Central Autonomic Network. These results provide insight into the underlying pathology of autonomic dysfunction in Huntington’s disease and its possible use as a marker for disease progression.

## Graphical Abstract

**Graphical abstract:**
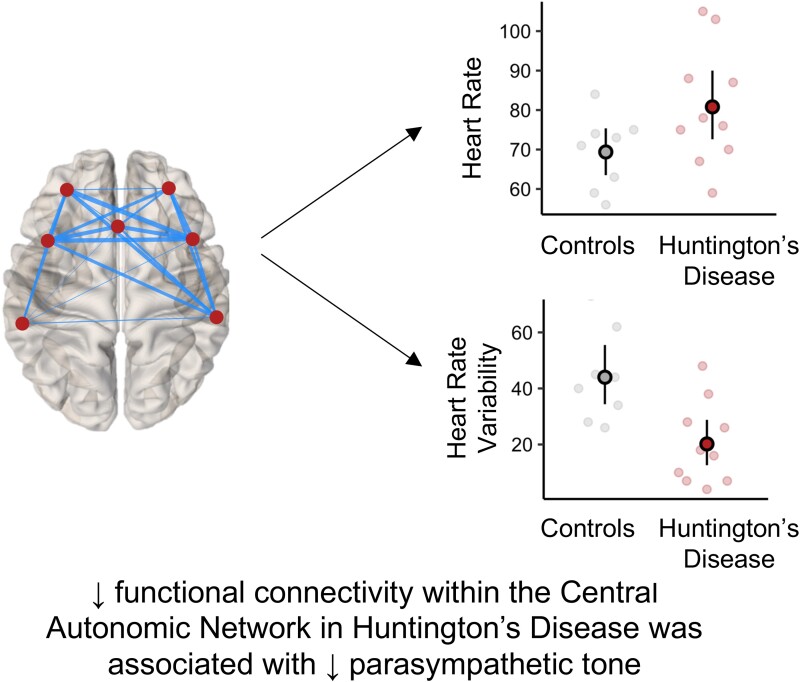

## Introduction

Huntington’s disease is a neurodegenerative disease caused by an abnormally high number of cytosine–adenine–guanine (CAG) repeats in the Huntingtin gene (*Htt*) encoding for the Huntingtin protein.^1,2^ Patients with Huntington’s disease experience progressively worsening motor, cognitive, and psychiatric symptoms that are associated with striatal degeneration.^3,4^ A relatively underinvestigated phenomenon associated with Huntington’s disease is an observed imbalance of the autonomic nervous system (ANS), with increased sympathetic tone.^5-9^ This increase in sympathetic tone may contribute to a number of important symptoms that significantly impact quality of life in Huntington’s disease, including sleep disturbances, sexual dysfunction, and bowel and bladder dysfunction.^8,10,11^ Evidence for increased sympathetic tone is seen in Huntington’s disease patients having elevated resting heart rate, blood pressure, and core body temperature relative to healthy individuals.^12,13^ The underlying cause of autonomic symptoms in Huntington’s disease is unknown. It has been hypothesized that the neurodegenerative nature of Huntington’s disease leads to dysfunction of brain regions that make up the central autonomic network (CAN) that is key in maintaining homeostatic balance of the ANS.^14^

The CAN is a network of brain regions consisting of the anterior insula (AI), anterior cingulate cortex (ACC), supramarginal gyri (SMG), the rostral prefrontal cortex (RPFC), along with the hypothalamus and autonomic nuclei in the brainstem.^15^ Functional MRI (fMRI) has been used to show that functional connectivity within the cortical CAN is correlated with cardiac autonomic functioning at rest.^16,17^ CAN activity, as measured by fMRI, may reflect underlying network dynamics by which the brain matches autonomic tone with demands imposed by changing circumstances in the environment. As such, connectivity in the CAN has the potential to relate to inter-individual differences in autonomic activity.

Neurodegenerative changes are known to begin prior to Huntington’s disease symptom onset. A recent study found that young adults with the gene mutation that causes Huntington’s disease who were ∼25 years from their predicted motor onset had significantly elevated concentrations of plasma neurofilament light chain protein compared with controls.^18^ Those results may indicate that the neurodegenerative changes observed in Huntington’s disease can be measured more than two decades before the onset of motor symptoms of Huntington’s disease. Our group also recently reported that children and adolescent carriers of the gene mutation that causes Huntington’s disease who were ∼25 years from their predicted motor onset of Huntington’s disease had significant elevations in heart rate, blood pressure, and core body temperature.^13^ We hypothesized that these changes in autonomic tone could be an early sign of neurodegeneration in Huntington’s disease that precedes motor symptoms by decades. Consequently, we performed a pilot study with two primary goals. First, we aimed to replicate previous findings indicating that patients with premanifest Huntington’s disease have a higher heart rate but with concomitantly decreased resting heart rate variability (HRV), elevated blood pressure, and elevated core body temperature relative to age- and sex-matched controls. Second, we set out to determine if patients with premanifest Huntington’s disease have decreased functional connectivity within the CAN, and if so, does the CAN connectivity correlate with autonomic tone, which would be consistent with the hypothesis of a central aetiology for autonomic disturbance in Huntington’s disease.

## Methods

### Participants

We recruited male participants with a CAG repeat ≥ 36 who had pre-motor-manifest Huntington’s disease and were between the ages of 18 and 65. Participants were recruited to the University of Iowa between 1 December 2018 and 3 March 2020. Given that this was a pilot study, only males were recruited to decrease confounding and avoid sex-specific differences in a small sample size. Specifically, participants had to have a diagnostic confidence level of less than four, per the Unified Huntington’s Disease Rating Scale (UHDRS) at the time of recruitment.^19^ Participants were excluded if they had a history of traumatic brain injury, history of stroke or myocardial infarction, history of chemotherapy, or if they were ineligible to undergo an MRI. A similar group of healthy, male comparison subjects was also recruited. In addition to the exclusion criteria listed above, healthy control subjects could not have a family history of Huntington’s disease, meaning they did not have a parent or grandparent with Huntington’s disease. Eligible participants underwent neuroimaging, an ECG to quantify heart rate variability, a motor assessment using the UHDRS, a functional assessment using the Total Functional Capacity score, and a cognitive assessment with the symbol digit modalities test. All participants provided written informed consent prior to participation in this study. The study protocol was approved by the Institutional Review Board at the University of Iowa (IRB ID#: 201810778) and was conducted in accordance with the Declaration of Helsinki.

We recruited 10 patients with premanifest Huntington’s disease and eight healthy comparison participants (Table 1). The groups were well-balanced on BMI. While there was not a statistically significant difference in age between the groups, the Huntington’s disease group was, on average, 8.12 years older than the control group. Older age is known to be associated with decreased parasympathetic tone and increased sympathetic tone,^20^ so this difference is an important consideration. Only one participant (from the pre-Huntington’s disease group) was using a an antihypertensive medication and no participants were using a stimulant medication (amphetamine-based, methylphenidate-based, or modafinil-based medication). The Huntington’s disease group was, on average, 14.59 years from their predicted motor onset.^21^ The Huntington’s disease group also did not differ from the comparison group in total motor score, total functional capacity, or cognitive function on the symbol digit modalities test, indicating that the Huntington’s disease group was presymptomatic.

**Table 1:** Baseline characteristics of participants

	Controls	Pre-HD	*P*-value
N	8	10	N/A
Age, mean ± SD	34.03 ± 13.66	42.15 ± 9.96	0.164
CAG, mean ± SD	N/A	41.20 ± 1.48	N/A
BMI, mean ± SD	28.70 ± 4.49	28.93 ± 5.78	0.927
Use of antihypertensive, N (%)	0 (0.00)	1 (10%)	1.00
Use of stimulant, N (%)	0 (0.00)	0 (0.00	1.00
UHDRS TMS	1.00 ± 2.14	3.50 ± 5.04	0.210
TFC	13.00 ± 0.00	12.30 ± 1.49	0.207
SDMT	51.00 ± 22.11	55.70 ± 13.07	0.581
One one	N/A	14.59 ± 7.75	N/A

Pre-HD, premanifest Huntington’s disease; SD, standard deviation; CAG, cytosine–adenine–guanine; BMI, body mass index; UHDRS TMS, Unified Huntington’s Disease Rating Scale Total Motor Score; TFC, total functional capacity; SDMT, Symbol Digit Modalities Test.

### Heart rate variability

All participants underwent a 3-lead ECG. Participants sat upright at rest and at least 5 min of uninterrupted ECG monitoring was performed by study personnel (AH or AB). The ECG was preprocessed using Kubios HRV Premium 3.5.0 Software.^22^ Specifically, automatic beat detection, noise detection, and beat correction were applied during preprocessing.^23^ Patients with Huntington’s disease have been hypothesized to have decreased parasympathetic tone.^6^ Therefore, the primary measures of heart rate variability used in this study was the time-domain parameters of root mean square of successive differences between normal heartbeats (rMSSD). rMSSD is less impacted by respiratory sinus arrhythmia and is the primary time-domain measure used to estimate HRV changes mediated by the parasympathetic nervous system.^24^ Secondary measures of ANS tone that were calculated included the log of the absolute power of the low frequency and high frequency bands, the ratio of low to high frequency bands (LF/HF), parasympathetic nervous system index (PNS index) and sympathetic nervous system index (SNS index). The PNS Index is calculated within the Kubios software using the parameters of mean R–R interval, rMSSD, and the Poincaré plot index SD1 in normalized units.^25^ The SNS index is also calculated within the Kubios software using mean heart rate, Baevsky’s stress index,^26^ and the Poincaré plot index SD2 in normalized units. All ECGs were reviewed for accuracy after preprocessing.

### Image acquisition and processing

Images were acquired on a research-dedicated 3 T GE scanner. Acquisition parameters for T_1_-weighted structural images were as follows: echo time = 2.496 ms; repetition time = 8.296 ms; inversion time = 1.0 s; flip angle = 8°; slice thickness = 0.8 mm; acquisition matrix = 320 × 320; field of view = 256 × 256 × 180 mm. The following parameters were used for acquisition of the functional images: echo time = 30 ms; repetition time = 2000 ms; flip angle = 80°; slice thickness = 4 mm; acquisitionmatrix = 64 × 64; field of view = 220 × 220 mm. Participants were instructed to keep their eyes closed, stay awake and not think of anything specific for ∼7 min.

Data were processed using the CONN toolbox^27^ for Matlab (version 2017a, licensed for use at the University of Iowa). For this analysis, we used the default preprocessing pipeline within the CONN toolbox, which included functional realignment and unwarping,^28^ slice timing correction, outlier identification, direct segmentation and normalization into stand stereotactic Montral Neurological Institute space,^29^ functional smoothing using spatial convolution with a Gaussian kernel of 8 mm full width half maximum, denoising with temporal band-pass filtering (0.008–0.09 Hz), linear detrending and further reduction of physiological noise using anatomical component-based noise reduction (aCompCor). We also performed motion scrubbing in the default processing pipeline to remove any sources of variance in outlier scans.^30^

#### Regions-of-interest

For this analysis, we were primarily interested in comparing functional connectivity within the CAN between the patients with Huntington’s disease and the healthy comparator subjects. The CAN was synonymous with the the default CONN network parcellation for the salience network, which was derived from an independent components analysis of 497 participants from the Human Connectome Project.^27^ In *post hoc* sensitivity analyses, we investigated functional connectivity in six additional networks defined in the default CONN network parcellation. Specifically, we investigated the visual network, sensorimotor network, dorsal attention network, frontoparietal network, default mode network (MD), and language network (Supplementary Table 2).

Pearson correlation coefficients representing the strength of functional connectivity between ROI’s were Fisher-transformed to Z-scores. The functional connectivity within each network was then quantified by taking the average connectivity across all ROIs within the network.^31^

### Statistical analysis

We first compared the vital sign data between the Huntington’s disease and healthy control groups using Analysis of Covariance (ANCOVA) models. ANCOVA models were also used to compare the primary (rMSSD) and secondary measures (HF, LF, LF/HF, PNS index, and SNS index) of heart rate variability between group. ANCOVA models were then constructed to compared the average connectivity within the nodes of the CAN and the other six networks, which served as tests of specificity for our hypothesized CAN-HRV relationship. For the analyses that included vital sign data or primary or secondary measures of HRV as the dependent variable, age and BMI were included as covariates. When the dependent variable was network connectivity, age was included as a coavariate since the impact of BMI on functional connectivity is not well-established. Lastly, we constructed a linear regression model with the primary HRV measure of rMSSD as the dependent variable to determine if average connectivity within the CAN predicted autonomic function amongst the patients with Huntington’s disease. Again, this models included age and BMI as covariates.

All analyses were carried out in RStudio, version 1.3.159. A *P*-value of <0.05 was considered statistically significant for all analyses.

### Data availability

De-identified clinical and imaging data may be made available upon reasonable request from qualified researchers.

## Results

**Figure 1:**
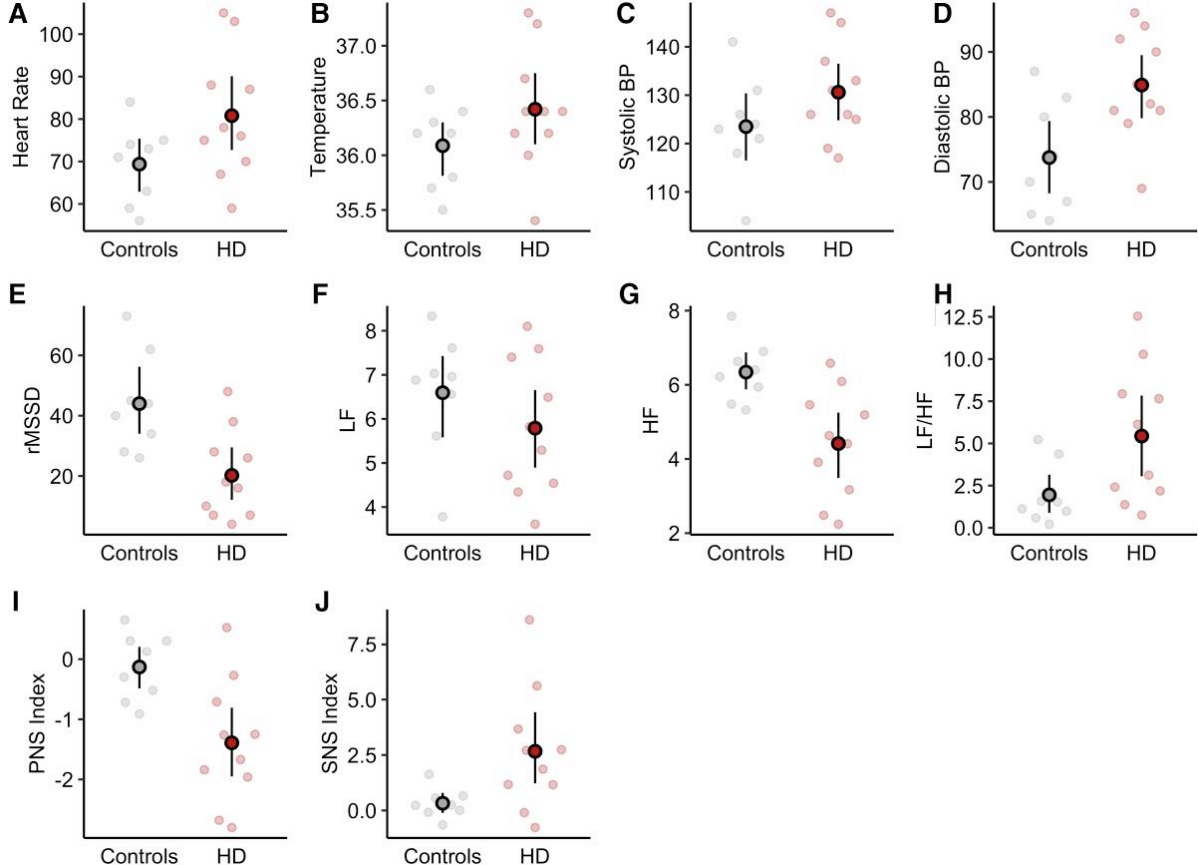
**Group differences in vital signs and autonomic measures.** The large dots represent the unadjusted means for each group and the associated lines represent the 95% confidence interval of the mean after constructing ANCOVA models controlling for age and BMI. The 95% confidence intervals of the mean differences between groups are presented in Table 2 (**A**) Group difference in heart rate: 13.30 bpm (*t* = −2.8, *P* = 0.020) (**B**) Group difference in core body temperature: 0.29 ° C (*t* = 2.14, *P* = 0.050) (**C**) Group difference in systolic BP: 8.05 mmHg (*t* = 2.50, 0.025) (**D**) Group difference in diastolic BP: 10.23 mmHg (*t* = 2.48, *P* = 0.026) (**E**) Group difference in rMSSD: −19.09 ms^2^ (*t* = −2.8, *P* = 0.014) (**F**) Group difference in LF: −0.40 log(ms) (*t* = −0.56, *P* = 0.588) (**G**) Group difference in HF: −1.57 log(ms) (*t* = −3.02, *P* = 0.009) (**H**) Group difference in LF/HF: 3.54 (*t* = 1.98, *P* = 0.068) (**I**) Group difference in PNS index: −1.26 SD (*t* = −3.59, *P* = 0.003) (**J**) Group difference in SNS index: 2.12 SD (*t* = 2.66, *P* = 0.019) BP, blood pressure; HF, Log of the high-frequency power band (log[ms]); LF, log of the low-frequency power band (log[ms]); LF/HF, ratio of LF to HF; PNS, parasympathetic nervous system; rMSSD, root mean square of successive differences between normal heartbeats (ms); SNS, sympathetic nervous system.

Compared with the healthy comparison group, the Huntington’s disease participants had significant elevations in resting heart rate, systolic blood pressure, and diastolic blood pressure. For the primary HRV outcome measure of rMSSD, the patients with Huntington’s disease also had significantly lower mean values compared with the healthy comparison group. The Huntington’s disease participants also had significantly lower power in the high frequency band but no differences in the low-frequency band. Lastly, the patients with Huntington’s disease had a significantly lower PNS index relative to the healthy comparator group and a significantly higher SNS index. These differences are outlined in Fig. 1 and Table 2. These results are in line with previous reports suggesting that patients with Huntington’s disease may have decreased parasympathetic tone that results in an increase in sympathetic tone.^6^ Importantly, all of these models included age as a covariate. This was especially important given the potential for age to impact vital signs and HRV and the approximate eight-year difference in age between the groups. However, age did not significantly predict any of the vital signs or HRV measures that were studied (Supplementary Table 1). This increases our confidence that the age difference between groups may not be significantly skewing these results. We performed an additional *post hoc* analysis to determine if the differences in HRV observed between the Huntington’s disease and control groups was due to their differences in age. Specifically, we compared the pre-Huntington’s disease group’s rMSSD value to published normative results using one-sample *t*-tests. The mean rSMMD value in males aged 40–49 years [similar to the mean age of the pre-Huntington’s disease group (42.15 years)] was approximately 34 ms.^32^ Another more recent study also found that the mean rMSSD value of 43 men aged 40–49 was also 34 ms.^33^ The pre-Huntington’s disease group in this study still had a significantly lower mean rMSSD values compared with both of these groups of healthy control males (*t* = −2.99, *p* = 0.015). Again, the results of this *post hoc* analysis increase our confidence that the observed changes in autonomic tone in the pre-Huntington’s disease group are the result of pathological changes rather than group differences induced by differences in age between the groups.

**Table 2:** Group differences in vital signs and heart rate variability measures

	Mean group difference (pre-HD-control)	95% Confidence interval	*P*-value
Resting heart rate	13.30	2.43–24.20	0.020
Systolic blood pressure	8.05	1.15–14.90	0.025
Diastolic blood pressure	10.23	1.40–19.10	0.026
Core body temperature	0.29	−0.001–0.574	0.050
rMSSD	−19.09	−33.70–−4.46	0.014
HF	−1.57	−2.69–−0.46	0.009
LF	−0.40	−1.93–1.14	0.588
LF/HF	3.54	−0.30–7.38	0.068
PNS index	−1.26	−2.01–−0.51	0.003
SNS index	2.12	0.41–3.84	0.019

HF, log of the high-frequency power band [log(ms)]; LF, log of the low-frequency power band [log(ms)]; LF/HF, ratio of LF to HF; PNS, parasympathetic nervous system; rMSSD, root mean square of successive differences between normal heartbeats (ms^2^); pre-HD, premanifest Huntington’s disease; SNS, sympathetic nervous system.

**Figure 2:**
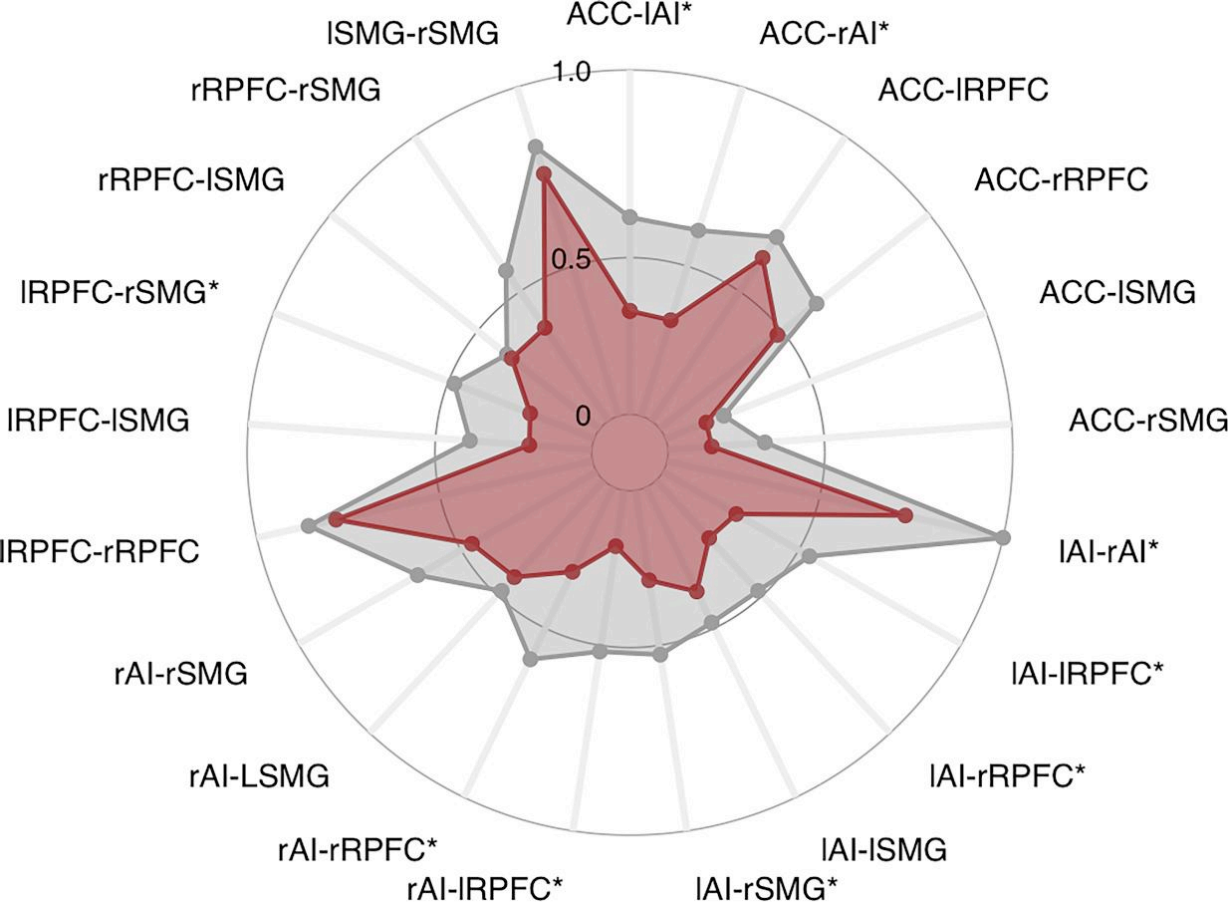
**Group differences in functional connectivity within the CAN.** The grey dots represent the mean functional connectivity *z*-score between the noted regions-of-interest in the healthy comparator group while the red dots represent the mean functional connectivity between regions-of-interest in the HD group. An asterisk next to the ROI-to-ROI indicates that the mean functional connectivity between those nodes differs significantly significantly (*P* < 0.05) between the groups based on independent samples *t*-tests. Details of the significant group differences are detailed below: Group difference in ACC-lAI: −0.30 (*t* = −2.93, *P* = 0.010) Group difference in ACC-rAI: −0.30 (*t* = −2.78, *P* = 0.013) Group difference in lAI-rAI: −0.32 (*t* = −2.69, *P* = 0.016) Group difference in lAI-lRPFC: −0.27 (*t* = −4.07, *P* = 0.001) Group difference in lAI-rRPFC: −0.23 (*t* = −2.34, *P* = 0.033) Group difference in lAI-rSMG: −0.24 (*t* = −2.72, *P* = 0.015) Group difference in rAI-lRPFC: −0.35 (*t* = −4.12, *P* = 0.001) Group difference in rAI-rRPFC: −0.31 (*t* = −2.78, *P* = 0.013) Group difference in lRPFC-rSM: −0.26 (*t* = −2.46, *P* = 0.026) ACC, anterior cingulate cortex; CAN, central autonomic network; lAI, left anterior insula; lRPFC, left rostral prefrontal cortex; lSMG, left supramarginal gyrus; rAI, right anterior insula; rRPFC, right rostral prefrontal cortex; rSMG, right supramarginal gyrus.

The mean functional connectivity within the CAN was significantly decreased in the Huntington’s disease group compared with the controls [MD = −0.38, 95% CI (−0.71–−0.06), *P* = 0.024; Fig. 2]. Patients with Huntington’s disease also had significantly lower mean functional connectivity within the default mode [MD = −0.15, 95% CI (−0.26–−0.05), *P* = 0.008] and the language network [MD = −0.23, 95% CI (−0.39–−0.07), *P* = 0.009]. There were no significant group differences within the visual, sensorimotor, dorsal attention, or frontoparietal networks.

Amongst participants in the Huntington’s disease group, there was a direct and significant relationship between mean functional connectivity within the CAN and rMSSD [Beta = 36.64, 95% CI (12.60–60.70), *P* = 0.009]. We also investigated the relationship between the mean functional connectivity of the language network and the default MD since the Huntington’s disease participants also had significantly decreased functional connectivity within these networks relative to the healthy comparator group. There were no statistically significant relationships between rMSSD and the mean functional connectivity within the default MD (*P* = 0.789) or the language network (*P* = 0.454).

## Discussion

Dysregulation of the ANS has been previously reported in patients with Huntington’s disease. However, it has not been clear from human studies what causes ANS dysfunction in patients with Huntington’s disease. Our current findings demonstrate that ANS dysfunction in Huntington’s disease is correlated with decreased functional connectivity within the CAN. While these results do not establish a direct causal link between dysfunction within the CAN and autonomic dysfunction in Huntington’s disease, they are consistent with the hypothesis that dysfunction of the ANS may be centrally mediated in Huntington’s disease.

Previous studies have demonstrated that functional connectivity within the CAN can influence ANS function,^34^ which is why we were particularly focused on this network. However, dysfunction of cortico-striatal networks in premanifest Huntington’s disease has been described previously. Therefore, we anticipated that decreased functional connectivity in other netowrks may be present as a consequence of the degenerative processes of Huntington’s disease. Decreased network connectivity was only observed, although, in the CAN, default MD, and language networks. Furthermore, HRV was only associated with functional connectivity within the CAN and not the default mode or language networks. These findings increase our confidence that the observed changes in HRV in the Huntington’s disease group are likely specific to changes within the CAN relative to other canonical networks.

Our group has previously reported that ANS dysfunction may be present as early as 25 years prior to the onset of motor symptoms in children who have inherited the gene that will cause Huntington’s disease. Specifically, in this previous study of children, the Huntington’s disease group had a mean resting heart rate that was ∼4 bpm higher than a healthy comparator group.^13^ In the present study, the Huntington’s disease group was, on average, closer to their predicted motor onset compared with the previous cohort of Huntington’s disease children. Furthermore, the group difference in resting heart rate in the present study was more pronounced than in our previous study with the Huntington’s disease group having a mean resting heart rate that was more than 10 bpm higher than the healthy comparator group. Therefore, these results may indicate that ANS dysfunction worsens as Huntington’s disease progresses. Regardless, ANS dysfunction seems to be a chronic feature of Huntington’s disease. Even mild elevations in sympathetic tone that are prolonged, as seems to be the case in Huntington’s disease, can have serious long-term consequences on cardiovascular and cerebrovascular health. Additionally, autonomic dysfunction in Huntington’s disease may be at the root of various symptoms that significantly impact quality of life in patients with Huntington’s disease.^8,10,11^ These results provide novel information about factors that are associated with ANS dysfunction in Huntington’s disease, which may lead to unique future treatment strategies for patients with Huntington’s disease.

There are important limitations to this study. First, the sample size was small. Second, the Huntington’s disease group was more than 8 years older than the control group, and older age is known to be associated with a decrease in parasympathetic tone and an increase sympathetic output. We attempted to control for this by including age as a covariate in all models. Furthermore, the effect of age was not found to be significantly influencing the models that compared vital signs and HRV between groups (Supplementary Table 1). Our *post hoc* analysis comparing the pre-Huntington’s disease group to healthy male controls of similar age also demonstrated that the pre-Huntington’s disease participants had significantly lower HRV compared with normative data from healthy controls. Regardless, the difference in age between groups should be considered when interpreting these results, especially in the setting of a small sample size. Previous studies of the ANS in patients with Huntington’s disease hypothesized that age and disease stage likely influence vital signs and HRV in Huntington’s disease.^35^ Given that the pre-Huntington’s disease group in our study was relatively far from their predicted motor onset of Huntington’s disease, the effect of age may have been decreased but may increase as participants’ disease burden increases. The last limitation of this study was the fact that it was cross-sectional, so it is unclear if the changes in functional connectivity and HRV are progressive. Future studies aimed at investigating the longitudinal nature of ANS changes in Huntington’s disease may help to further demonstrate the usefulness of the ANS as a marker of disease-related progression.

In conclusion, we have replicated previous findings demonstrating that patients with Huntington’s disease seem to have an imbalance in ANS tone that results in decreased heart rate variability, increased resting heart rate and increased blood pressure. These observed indications of ANS dysfunction were associated with decreased functional connectivity within the CAN in patients with Huntington’s disease. These results provide further insight into the possible underlying pathology of ANS dysfunction in patients with Huntington’s disease.

## Supplementary Material

fcac253_Supplementary_Data

## Data Availability

De-identified clinical and imaging data may be made available upon reasonable request from qualified researchers.

## Abbreviations

ACC =: anterior cingulate cortex
AI =: anterior insula
ANS =: autonomic nervous system
BMI =: body mass index
CAN =: central autonomic network
fMRI =: functional MRI
HRV =: heart rate variability
PNS =: parasympathetic nervous system
rMSSD =: root mean square of successive differences between normal heartbeats
RPFC =: rostral prefrontal cortex
SMG =: supramarginal gyri
SNS =: sympathetic nervous system
UHDRS =: Unified Huntington’s Disease Rating Scale
