# Supplementary material for: Autonomic changes in Huntington’s disease correlate with altered central autonomic network connectivity
Source: Brain Commun. 2022 Oct 7;4(5):fcac253. doi: 10.1093/braincomms/fcac253 (PMC9617256; doi:10.1093/braincomms/fcac253)
Supplement: fcac253_Supplementary_Data [file fcac253_supplementary_data.docx]

**Supplementary Table 1: Effect of Age on Vital Signs and Heart Rate Variability**

| **Variable** | **Coefficient** | **95% Confidence Interval** | **T-Value** | **p-value** |
| --- | --- | --- | --- | --- |
| **Resting Heart Rate** | -0.27 | -0.76 – 0.22 | -1.19 | 0.255 |
| **Systolic Blood Pressure** | -0.16 | -0.47 – 0.15 | -1.09 | 0.29 |
| **Diastolic Blood Pressure** | 0.09 | -0.31 – 0.49 | 0.49 | 0.631 |
| **Core Body Temperature** | 0.00 | -0.01 – 0.02 | 0.54 | 0.599 |
| **rMSSD** | -0.53 | -1.19 – 0.13 | -1.73 | 0.106 |
| **HF** | -0.04 | -0.09 – 0.01 | -1.67 | 0.118 |
| **LF** | -0.05 | -0.12 – 0.02 | -1.45 | 0.169 |
| **LF/HF** | -0.01 | -0.18 – 0.17 | -0.09 | 0.928 |
| **PNS Index** | 0.00 | -0.03 – 0.04 | 0.19 | 0.855 |
| **SNS Index** | 0.02 | -0.06 – 0.10 | 0.50 | 0.624 |

HF: Log of the high frequency power band (log[ms])

LF: Log of the low frequency power band (log[ms])

LF/HF: Ratio of LF to HF

PNS: Parasympathetic nervous system

rMSSD: Root mean square of successive differences between normal heartbeats (ms)

SNS: Sympathetic nervous system

**Supplementary Table 2: Network Composition**

|  | **ROI** | **X** | **Y** | **Z** |
| --- | --- | --- | --- | --- |
| Central Autonomic Network | Anterior Cingulate Cortex | 0 | 22 | 35 |
|  | Left Anterior Insula | -44 | 13 | 1 |
|  | Right Anterior Insula | 47 | 14 | 0 |
|  | Left Rostral Prefrontal Cortex | -32 | 45 | 27 |
|  | Right Rostral Prefrontal Cortex | 32 | 46 | 27 |
|  | Left Supramarginal Gyrus | -60 | -39 | 31 |
|  | Right Supramarginal Gyrus | 62 | -35 | 32 |
| Visual Network | Medial Visual | 2 | -79 | 12 |
|  | Occipital Visual | 0 | -93 | -4 |
|  | Left Lateral Visual | -37 | -79 | 10 |
|  | Right Lateral Visual | 38 | -72 | 13 |
| Sensorimotor Network | Left Lateral Motor | -55 | -12 | 29 |
|  | Right Lateral Motor | 56 | -10 | 29 |
|  | Superior Motor | 0 | -31 | 67 |
| Dorsal Attention Network | Left Frontal Eye Fields | -27 | -9 | 64 |
|  | Right Frontal Eye Fields | 30 | -6 | 64 |
|  | Left Intraparietal Sulcus | -39 | -43 | 52 |
|  | Right Intraparietal Sulcus | 39 | -42 | 54 |
| Fronto-Parietal Network | Left Lateral Prefrontal Cortex | -43 | 33 | 28 |
|  | Left Posterior Parietal Cortex | -46 | -58 | 49 |
|  | Right Lateral Prefrontal Cortex | 41 | 38 | 30 |
|  | Right Posterior Parietal Cortex | 52 | -52 | 45 |
| Default Mode Network | Medial Prefrontal Cortex | 1 | 55 | -3 |
|  | Left Lateral Parietal | -39 | -77 | 33 |
|  | Right Lateral Parietal | 47 | -67 | 29 |
|  | Posterior Cingulate Cortex | 1 | -61 | 38 |
| Language Network | Left Inferior Frontal Gyrus | -51 | 26 | 2 |
|  | Right Interior Frontal Gyrus | 54 | 28 | 1 |
|  | Left Posterior Superior Temporal Gyrus | -57 | -47 | 15 |
|  | Right Posterior Superior Temporal Gyrus | 59 | -42 | 13 |
